# (*E*)-1,3-Dimethyl-2,6-di­phenyl­piperidin-4-one *O*-(phen­oxy­carbon­yl)oxime

**DOI:** 10.1107/S1600536814010526

**Published:** 2014-05-24

**Authors:** B. Raghuvarman, R. Sivakumar, K. Gokula Krishnan, V. Thanikachalam, S. Aravindhan

**Affiliations:** a Department of Physics, Presidency College, Chennai 600 005, India; b Department Of Chemistry, Annamalai University, Annamalai Nagar 608 002, India

## Abstract

The title piperidine derivative, C_26_H_26_N_2_O_3_, has an *E* conformation about the N=C bond. The piperidine ring has a chair conformation and its mean plane is almost perpendicular to the attached phenyl rings, making dihedral angles of 87.47 (9) and 87.34 (8)°. The planes of these two phenyl rings are inclined to one another by 60.38 (9)°. The plane of the terminal phenyl ring is tilted at an angle of 32.79 (9)° to the mean plane of the piperidine ring. The mol­ecular conformation is stabilized by two intra­molecular C—H⋯O contacts. There are no significant inter­molecular inter­actions in the crystal.

## Related literature   

For the biological activity of piperidine derivatives, see, for example: Moldt *et al.* (1997 ▶); Peters *et al.* (2009 ▶). For asymmetry parameters, see: Nardelli (1983 ▶).
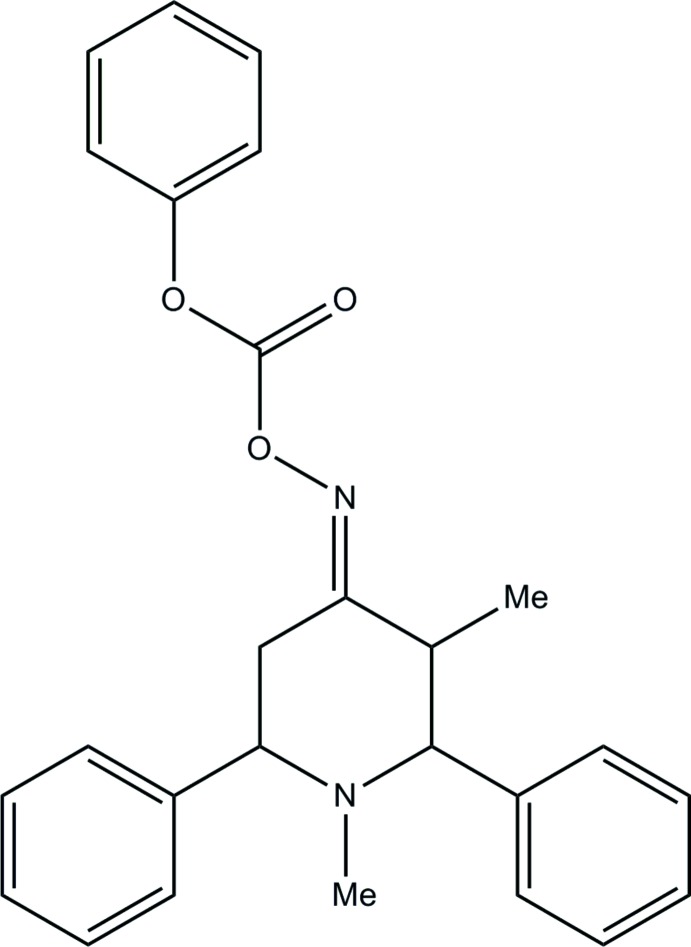


## Experimental   

### 

#### Crystal data  

C_26_H_26_N_2_O_3_
*M* *_r_* = 414.49
Monoclinic, 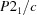

*a* = 16.2004 (12) Å
*b* = 11.9587 (10) Å
*c* = 11.3601 (7) Å
β = 102.547 (2)°
*V* = 2148.3 (3) Å^3^
*Z* = 4
Mo *K*α radiation
μ = 0.08 mm^−1^
*T* = 293 K
0.25 × 0.20 × 0.20 mm

#### Data collection  

Bruker Kappa APEXII CCD diffractometer
Absorption correction: multi-scan (*SADABS*; Bruker 2004 ▶) *T*_min_ = 0.979, *T*_max_ = 0.983
27566 measured reflections
6686 independent reflections
3950 reflections with *I* > 2σ(*I*)
*R*_int_ = 0.033

#### Refinement  

*R*[*F* ^2^ > 2σ(*F* ^2^)] = 0.053
*wR*(*F* ^2^) = 0.180
*S* = 1.01
6686 reflections
282 parameters
H-atom parameters constrained
Δρ_max_ = 0.33 e Å^−3^
Δρ_min_ = −0.35 e Å^−3^

### 

Data collection: *APEX2* (Bruker, 2004 ▶); cell refinement: *APEX2*/*SAINT* (Bruker, 2004 ▶); data reduction: *SAINT*/*XPREP* ; program(s) used to solve structure: *SHELXS97* (Sheldrick, 2008 ▶); program(s) used to refine structure: *SHELXL97* (Sheldrick, 2008 ▶); molecular graphics: *ORTEP-3 for Windows* (Farrugia, 2012 ▶); software used to prepare material for publication: *SHELXL97* and *PLATON* (Spek, 2009 ▶).

## Supplementary Material

Crystal structure: contains datablock(s) I, global. DOI: 10.1107/S1600536814010526/su2678sup1.cif

Structure factors: contains datablock(s) I. DOI: 10.1107/S1600536814010526/su2678Isup2.hkl

Supporting information file. DOI: 10.1107/S1600536814010526/su2678Isup3.cml

CCDC reference: 1001814

Additional supporting information:  crystallographic information; 3D view; checkCIF report

## Figures and Tables

**Table 1:** Hydrogen-bond geometry (Å, °)

*D*—H⋯*A*	*D*—H	H⋯*A*	*D*⋯*A*	*D*—H⋯*A*
C3—H3*A*⋯O1	0.97	2.27	2.6881 (18)	105
C26—H26⋯O2	0.93	2.30	2.823 (2)	115

**Fig. 1.**
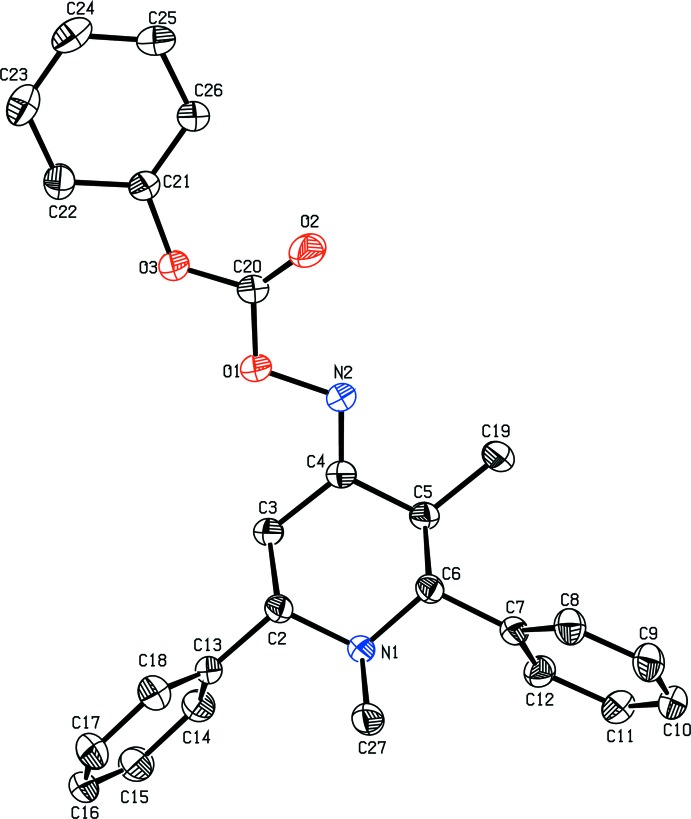
The molecular structure of the title molecule, with atom labelling. Displacement ellipsoids are drawn at the 30% probability level. H atoms have been omitted for clarity.

**Fig. 2.**
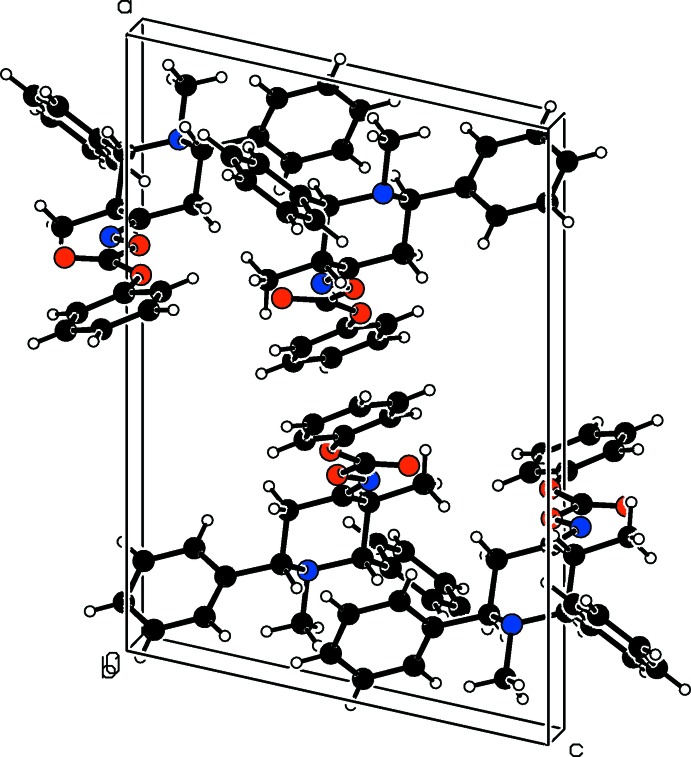
A view along the b axis of the crystal packing of the title compound.

## supplementary crystallographic information

### 1. Comment

In view of the biological importance of piperidine derivatives (Moldt *et al.*, 1997; Peters *et al.*, 2009) we undertook the synthesis of the title compound and report herein on its crystal structure.

In the title molecule, Fig. 1, the piperidine ring (N1/C2—C6) [DS (C3) = 0.005 (8) Å and D2 (C3—C2) = 0.015 (6) Å] adopts a chair conformation defined by the above asymmetry parameters (Nardelli, 1983). Its mean plane is almost perpendicular to the attached phenyl rings (C13—C18 and C7—C12), with dihedral angles of 87.47 (9)° and 87.34 (8)°, respectively. These two phenyl rings are inclined to one another by 60.38 (9) °. More over the mean plane of the piperidine ring is tilted by 32.79 (9)° with respect to the terminal phenyl ring (C21-C26). The molecular conformation is stabilized by two intramolecular C-H···O contacts (Table 1).

In the crystal, there are no significant intermolecular interactions present (Fig. 2).

### 2. Experimental

The title compound was synthesized by Mannich condensation. Benzaldehyde (2 mol), ammonium acetate (1 mol) and ethyl methyl ketone (1 mol) in absolute ethanol were warmed for 30 min and then stirred overnight at room temperature. The product obtained was treated with methyl iodide (1.5 mol) in the presence of potassium carbonate (2 mol) in acetone (10 ml) and the mixture was refluxed to give 1,3-dimethyl-2,6-diphenylpiperidin-4-one (I). Oximation was carried out by refluxing (I) with hydroxylamine hydrochloride (2 mol) in the presence of sodium acetate (2 mol) in ethanol (10 ml). To the resulting oxime (0.5 g, 1.79 mmol) in dry tetrahydrofuran (10 ml), was added potassium carbonate (0.48 g, 3.52 mmol) followed by tetrabutylammonium bromide (0.58 g,1.79 mmol). After stirring for 15 min, phenyl chloroformate (0.38 g, 2.68 mmol) was added drop wise over a period of 15 min. The mixture was stirred at ambient temperature for 2 h and progress of the reaction was monitored by thin layer chromatography. Upon completion of the reaction, the mixture was diluted with water (20 ml) and extracted with dichloromethane (2 × 20 ml). The combined organic layers were washed with water (2 × 20 ml), then brine solution (20 ml), and then dried over anhydrous sodium sulfate (5 g). The mixture was the filtered and concentrated under reduced pressure. The crude product obtained was purified by column chromatography over silica gel (100–200 mesh) eluted with a solvent system of ethyl acetate:petroleum ether (2:98). The pure fractions were collected and concentrated under reduced pressure giving a white solid (0.63 g, 85%). This was recrystallized from a DMF-water mixture (9:1) to yield colourless block-like crystals suitable for X-ray diffraction studies.

### 3. Refinement

All the H atoms were positioned geometrically and constrained to ride on their parent atom: C–H = 0.93–0.98 Å with *U*_iso_(H) = 1.5U_eq_(C-methyl) and = 1.2U_eq_(C) for other H atoms.

### Crystal data

**Table:**

C_26_H_26_N_2_O_3_	*F*(000) = 880
*M**_r_* = 414.49	*D*_x_ = 1.282 Mg m^−^^3^
Monoclinic, *P*2_1_/*c*	Mo *K*α radiation, λ = 0.71073 Å
Hall symbol: -P 2ybc	Cell parameters from 8834 reflections
*a* = 16.2004 (12) Å	θ = 2.1–31.2°
*b* = 11.9587 (10) Å	µ = 0.08 mm^−^^1^
*c* = 11.3601 (7) Å	*T* = 293 K
β = 102.547 (2)°	Block, colourless
*V* = 2148.3 (3) Å^3^	0.25 × 0.20 × 0.20 mm
*Z* = 4	

### Data collection

**Table:**

Bruker Kappa APEXII CCD diffractometer	6686 independent reflections
Radiation source: fine-focus sealed tube	3950 reflections with *I* > 2σ(*I*)
Graphite monochromator	*R*_int_ = 0.033
ω and φ scan	θ_max_ = 30.8°, θ_min_ = 2.5°
Absorption correction: multi-scan (*SADABS*; Bruker 2004)	*h* = −23→21
*T*_min_ = 0.979, *T*_max_ = 0.983	*k* = −17→17
27566 measured reflections	*l* = −16→16

### Refinement

**Table:**

Refinement on *F*^2^	Primary atom site location: structure-invariant direct methods
Least-squares matrix: full	Secondary atom site location: difference Fourier map
*R*[*F*^2^ > 2σ(*F*^2^)] = 0.053	Hydrogen site location: inferred from neighbouring sites
*wR*(*F*^2^) = 0.180	H-atom parameters constrained
*S* = 1.01	*w* = 1/[σ^2^(*F*_o_^2^) + (0.093*P*)^2^ + 0.2301*P*] where *P* = (*F*_o_^2^ + 2*F*_c_^2^)/3
6686 reflections	(Δ/σ)_max_ < 0.001
282 parameters	Δρ_max_ = 0.33 e Å^−^^3^
0 restraints	Δρ_min_ = −0.35 e Å^−^^3^

### Special details

**Table:**

Geometry. All e.s.d.'s (except the e.s.d. in the dihedral angle between two l.s. planes) are estimated using the full covariance matrix. The cell e.s.d.'s are taken into account individually in the estimation of e.s.d.'s in distances, angles and torsion angles; correlations between e.s.d.'s in cell parameters are only used when they are defined by crystal symmetry. An approximate (isotropic) treatment of cell e.s.d.'s is used for estimating e.s.d.'s involving l.s. planes.
Refinement. Refinement of *F*^2^ against ALL reflections. The weighted *R*-factor *wR* and goodness of fit *S* are based on *F*^2^, conventional *R*-factors *R* are based on *F*, with *F* set to zero for negative *F*^2^. The threshold expression of *F*^2^ > σ(*F*^2^) is used only for calculating *R*-factors(gt) *etc*. and is not relevant to the choice of reflections for refinement. *R*-factors based on *F*^2^ are statistically about twice as large as those based on *F*, and *R*- factors based on ALL data will be even larger.

### Fractional atomic coordinates and isotropic or equivalent isotropic displacement parameters (Å^2^)

**Table:**

	*x*	*y*	*z*	*U*_iso_*/*U*_eq_	
C2	0.16989 (9)	0.35382 (12)	0.83490 (12)	0.0379 (3)	
H2	0.1382	0.2997	0.8726	0.045*	
C3	0.26100 (11)	0.31168 (15)	0.85152 (13)	0.0467 (4)	
H3A	0.2608	0.2355	0.8220	0.056*	
H3B	0.2915	0.3579	0.8051	0.056*	
C4	0.30428 (9)	0.31542 (13)	0.98144 (13)	0.0393 (3)	
C5	0.30006 (9)	0.42707 (12)	1.03942 (13)	0.0389 (3)	
H5	0.3253	0.4815	0.9933	0.047*	
C6	0.20588 (9)	0.45817 (12)	1.02319 (12)	0.0368 (3)	
H6	0.1778	0.3995	1.0603	0.044*	
C7	0.19662 (9)	0.56761 (13)	1.08548 (12)	0.0382 (3)	
C8	0.15971 (11)	0.57068 (15)	1.18468 (14)	0.0505 (4)	
H8	0.1385	0.5053	1.2112	0.061*	
C9	0.15411 (12)	0.67029 (18)	1.24476 (15)	0.0590 (5)	
H9	0.1293	0.6711	1.3113	0.071*	
C10	0.18471 (12)	0.76735 (16)	1.20726 (15)	0.0565 (5)	
H10	0.1806	0.8340	1.2477	0.068*	
C11	0.22174 (12)	0.76556 (15)	1.10914 (16)	0.0529 (4)	
H11	0.2430	0.8312	1.0833	0.064*	
C12	0.22743 (11)	0.66632 (14)	1.04893 (14)	0.0455 (4)	
H12	0.2525	0.6661	0.9826	0.055*	
C13	0.12923 (10)	0.35957 (13)	0.70176 (12)	0.0399 (3)	
C14	0.16171 (12)	0.42803 (15)	0.62530 (14)	0.0509 (4)	
H14	0.2105	0.4692	0.6550	0.061*	
C15	0.12225 (15)	0.43587 (17)	0.50478 (15)	0.0654 (6)	
H15	0.1445	0.4822	0.4537	0.078*	
C16	0.05001 (15)	0.37504 (19)	0.46032 (16)	0.0700 (6)	
H16	0.0228	0.3817	0.3796	0.084*	
C17	0.01834 (14)	0.30530 (19)	0.53423 (18)	0.0689 (6)	
H17	−0.0298	0.2630	0.5038	0.083*	
C18	0.05790 (12)	0.29753 (16)	0.65455 (16)	0.0545 (4)	
H18	0.0361	0.2496	0.7047	0.065*	
C19	0.34923 (12)	0.43356 (15)	1.16909 (15)	0.0546 (4)	
H19A	0.3214	0.3891	1.2193	0.082*	
H19B	0.3519	0.5099	1.1958	0.082*	
H19C	0.4055	0.4058	1.1743	0.082*	
C20	0.37323 (10)	0.05039 (13)	1.04333 (14)	0.0445 (4)	
C21	0.41582 (10)	−0.13514 (13)	1.00076 (14)	0.0446 (4)	
C22	0.39797 (12)	−0.21341 (15)	0.91128 (15)	0.0534 (4)	
H22	0.3654	−0.1951	0.8358	0.064*	
C23	0.42925 (14)	−0.32043 (16)	0.93527 (19)	0.0640 (5)	
H23	0.4181	−0.3746	0.8752	0.077*	
C24	0.47672 (13)	−0.34739 (16)	1.04712 (19)	0.0619 (5)	
H24	0.4969	−0.4199	1.0630	0.074*	
C25	0.49410 (13)	−0.26815 (16)	1.13426 (18)	0.0622 (5)	
H25	0.5262	−0.2866	1.2100	0.075*	
C26	0.46459 (12)	−0.16009 (15)	1.11162 (16)	0.0587 (5)	
H26	0.4777	−0.1053	1.1708	0.070*	
C27	0.07667 (10)	0.49575 (17)	0.87840 (15)	0.0533 (4)	
H27A	0.0505	0.4981	0.7941	0.080*	
H27B	0.0732	0.5682	0.9134	0.080*	
H27C	0.0480	0.4420	0.9180	0.080*	
N1	0.16563 (7)	0.46358 (10)	0.89327 (10)	0.0357 (3)	
N2	0.34143 (8)	0.23699 (11)	1.04748 (11)	0.0438 (3)	
O1	0.34161 (8)	0.13746 (10)	0.97534 (9)	0.0528 (3)	
O2	0.38576 (11)	0.04349 (12)	1.14880 (12)	0.0795 (5)	
O3	0.38400 (9)	−0.02830 (10)	0.96515 (10)	0.0643 (4)	

### Atomic displacement parameters (Å^2^)

**Table:**

	*U*^11^	*U*^22^	*U*^33^	*U*^12^	*U*^13^	*U*^23^
C2	0.0455 (8)	0.0346 (7)	0.0319 (6)	0.0009 (6)	0.0048 (6)	0.0017 (5)
C3	0.0558 (10)	0.0460 (9)	0.0356 (7)	0.0169 (7)	0.0042 (6)	0.0000 (6)
C4	0.0394 (7)	0.0390 (8)	0.0379 (7)	0.0063 (6)	0.0054 (6)	0.0017 (6)
C5	0.0430 (8)	0.0345 (7)	0.0369 (7)	0.0018 (6)	0.0035 (6)	0.0030 (6)
C6	0.0432 (8)	0.0374 (8)	0.0297 (6)	0.0003 (6)	0.0077 (5)	0.0022 (5)
C7	0.0403 (8)	0.0421 (8)	0.0315 (6)	0.0047 (6)	0.0061 (5)	−0.0001 (6)
C8	0.0569 (10)	0.0568 (11)	0.0406 (8)	−0.0011 (8)	0.0171 (7)	−0.0023 (7)
C9	0.0672 (12)	0.0733 (13)	0.0403 (8)	0.0103 (10)	0.0200 (8)	−0.0092 (8)
C10	0.0633 (11)	0.0537 (11)	0.0486 (9)	0.0150 (9)	0.0039 (8)	−0.0141 (8)
C11	0.0594 (10)	0.0407 (9)	0.0575 (10)	0.0038 (8)	0.0101 (8)	−0.0021 (7)
C12	0.0530 (9)	0.0439 (9)	0.0417 (8)	0.0065 (7)	0.0147 (7)	0.0011 (7)
C13	0.0472 (8)	0.0369 (8)	0.0330 (6)	0.0077 (6)	0.0033 (6)	−0.0026 (6)
C14	0.0631 (11)	0.0490 (10)	0.0403 (8)	0.0061 (8)	0.0106 (7)	0.0045 (7)
C15	0.0961 (16)	0.0622 (12)	0.0386 (8)	0.0197 (11)	0.0164 (9)	0.0081 (8)
C16	0.0919 (15)	0.0724 (14)	0.0355 (8)	0.0366 (12)	−0.0086 (9)	−0.0114 (9)
C17	0.0645 (12)	0.0750 (14)	0.0556 (11)	0.0092 (10)	−0.0126 (9)	−0.0179 (10)
C18	0.0552 (10)	0.0560 (11)	0.0483 (9)	−0.0004 (8)	0.0024 (7)	−0.0042 (8)
C19	0.0599 (10)	0.0488 (10)	0.0455 (8)	0.0038 (8)	−0.0094 (7)	−0.0011 (7)
C20	0.0463 (9)	0.0403 (8)	0.0439 (8)	0.0077 (7)	0.0033 (6)	0.0008 (6)
C21	0.0487 (9)	0.0367 (8)	0.0461 (8)	0.0044 (7)	0.0051 (7)	0.0002 (7)
C22	0.0631 (11)	0.0483 (10)	0.0477 (9)	0.0000 (8)	0.0097 (8)	−0.0060 (8)
C23	0.0785 (13)	0.0450 (10)	0.0721 (12)	−0.0025 (9)	0.0240 (10)	−0.0132 (9)
C24	0.0689 (12)	0.0390 (9)	0.0833 (14)	0.0092 (9)	0.0283 (10)	0.0044 (9)
C25	0.0678 (12)	0.0529 (11)	0.0615 (11)	0.0182 (9)	0.0044 (9)	0.0091 (9)
C26	0.0675 (11)	0.0477 (10)	0.0521 (9)	0.0146 (8)	−0.0066 (8)	−0.0046 (8)
C27	0.0437 (9)	0.0664 (12)	0.0465 (9)	0.0141 (8)	0.0029 (7)	−0.0087 (8)
N1	0.0373 (6)	0.0380 (6)	0.0306 (5)	0.0054 (5)	0.0046 (4)	−0.0008 (5)
N2	0.0496 (7)	0.0368 (7)	0.0420 (7)	0.0081 (6)	0.0037 (5)	−0.0018 (5)
O1	0.0691 (8)	0.0402 (6)	0.0433 (6)	0.0174 (6)	−0.0004 (5)	0.0004 (5)
O2	0.1305 (14)	0.0596 (9)	0.0476 (7)	0.0390 (9)	0.0178 (8)	0.0083 (6)
O3	0.0964 (10)	0.0428 (7)	0.0440 (6)	0.0213 (7)	−0.0060 (6)	−0.0031 (5)

### Geometric parameters (Å, º)

**Table:**

C2—N1	1.4790 (18)	C15—C16	1.377 (3)
C2—C13	1.5144 (18)	C15—H15	0.9300
C2—C3	1.532 (2)	C16—C17	1.360 (3)
C2—H2	0.9800	C16—H16	0.9300
C3—C4	1.491 (2)	C17—C18	1.381 (2)
C3—H3A	0.9700	C17—H17	0.9300
C3—H3B	0.9700	C18—H18	0.9300
C4—N2	1.2683 (19)	C19—H19A	0.9600
C4—C5	1.497 (2)	C19—H19B	0.9600
C5—C19	1.517 (2)	C19—H19C	0.9600
C5—C6	1.542 (2)	C20—O2	1.1738 (19)
C5—H5	0.9800	C20—O1	1.3310 (18)
C6—N1	1.4797 (17)	C20—O3	1.3312 (19)
C6—C7	1.511 (2)	C21—C22	1.366 (2)
C6—H6	0.9800	C21—C26	1.366 (2)
C7—C12	1.380 (2)	C21—O3	1.4040 (19)
C7—C8	1.386 (2)	C22—C23	1.382 (3)
C8—C9	1.386 (3)	C22—H22	0.9300
C8—H8	0.9300	C23—C24	1.373 (3)
C9—C10	1.366 (3)	C23—H23	0.9300
C9—H9	0.9300	C24—C25	1.355 (3)
C10—C11	1.376 (3)	C24—H24	0.9300
C10—H10	0.9300	C25—C26	1.382 (2)
C11—C12	1.383 (2)	C25—H25	0.9300
C11—H11	0.9300	C26—H26	0.9300
C12—H12	0.9300	C27—N1	1.4655 (19)
C13—C14	1.378 (2)	C27—H27A	0.9600
C13—C18	1.380 (2)	C27—H27B	0.9600
C14—C15	1.383 (2)	C27—H27C	0.9600
C14—H14	0.9300	N2—O1	1.4454 (16)
			
N1—C2—C13	110.79 (11)	C16—C15—C14	120.03 (19)
N1—C2—C3	111.98 (12)	C16—C15—H15	120.0
C13—C2—C3	109.65 (12)	C14—C15—H15	120.0
N1—C2—H2	108.1	C17—C16—C15	120.12 (16)
C13—C2—H2	108.1	C17—C16—H16	119.9
C3—C2—H2	108.1	C15—C16—H16	119.9
C4—C3—C2	110.21 (12)	C16—C17—C18	119.7 (2)
C4—C3—H3A	109.6	C16—C17—H17	120.1
C2—C3—H3A	109.6	C18—C17—H17	120.1
C4—C3—H3B	109.6	C13—C18—C17	121.20 (19)
C2—C3—H3B	109.6	C13—C18—H18	119.4
H3A—C3—H3B	108.1	C17—C18—H18	119.4
N2—C4—C3	128.62 (14)	C5—C19—H19A	109.5
N2—C4—C5	117.42 (13)	C5—C19—H19B	109.5
C3—C4—C5	113.94 (12)	H19A—C19—H19B	109.5
C4—C5—C19	114.10 (12)	C5—C19—H19C	109.5
C4—C5—C6	107.53 (12)	H19A—C19—H19C	109.5
C19—C5—C6	113.64 (13)	H19B—C19—H19C	109.5
C4—C5—H5	107.1	O2—C20—O1	127.35 (15)
C19—C5—H5	107.1	O2—C20—O3	127.78 (15)
C6—C5—H5	107.1	O1—C20—O3	104.81 (13)
N1—C6—C7	111.23 (11)	C22—C21—C26	121.53 (16)
N1—C6—C5	109.87 (11)	C22—C21—O3	113.84 (14)
C7—C6—C5	110.45 (12)	C26—C21—O3	124.53 (15)
N1—C6—H6	108.4	C21—C22—C23	118.60 (17)
C7—C6—H6	108.4	C21—C22—H22	120.7
C5—C6—H6	108.4	C23—C22—H22	120.7
C12—C7—C8	118.03 (14)	C24—C23—C22	120.51 (18)
C12—C7—C6	121.39 (13)	C24—C23—H23	119.7
C8—C7—C6	120.53 (14)	C22—C23—H23	119.7
C9—C8—C7	120.60 (17)	C25—C24—C23	119.84 (18)
C9—C8—H8	119.7	C25—C24—H24	120.1
C7—C8—H8	119.7	C23—C24—H24	120.1
C10—C9—C8	120.66 (16)	C24—C25—C26	120.61 (18)
C10—C9—H9	119.7	C24—C25—H25	119.7
C8—C9—H9	119.7	C26—C25—H25	119.7
C9—C10—C11	119.40 (16)	C21—C26—C25	118.88 (17)
C9—C10—H10	120.3	C21—C26—H26	120.6
C11—C10—H10	120.3	C25—C26—H26	120.6
C10—C11—C12	120.11 (17)	N1—C27—H27A	109.5
C10—C11—H11	119.9	N1—C27—H27B	109.5
C12—C11—H11	119.9	H27A—C27—H27B	109.5
C7—C12—C11	121.19 (15)	N1—C27—H27C	109.5
C7—C12—H12	119.4	H27A—C27—H27C	109.5
C11—C12—H12	119.4	H27B—C27—H27C	109.5
C14—C13—C18	118.45 (14)	C27—N1—C2	108.77 (12)
C14—C13—C2	120.91 (14)	C27—N1—C6	109.62 (11)
C18—C13—C2	120.63 (14)	C2—N1—C6	110.79 (11)
C13—C14—C15	120.44 (18)	C4—N2—O1	109.43 (11)
C13—C14—H14	119.8	C20—O1—N2	111.29 (11)
C15—C14—H14	119.8	C20—O3—C21	122.97 (13)
			
N1—C2—C3—C4	51.47 (17)	C14—C15—C16—C17	−1.4 (3)
C13—C2—C3—C4	174.87 (13)	C15—C16—C17—C18	1.4 (3)
C2—C3—C4—N2	125.06 (18)	C14—C13—C18—C17	−1.5 (3)
C2—C3—C4—C5	−53.16 (18)	C2—C13—C18—C17	177.48 (16)
N2—C4—C5—C19	5.7 (2)	C16—C17—C18—C13	0.1 (3)
C3—C4—C5—C19	−175.91 (14)	C26—C21—C22—C23	1.0 (3)
N2—C4—C5—C6	−121.35 (15)	O3—C21—C22—C23	177.45 (17)
C3—C4—C5—C6	57.08 (16)	C21—C22—C23—C24	0.5 (3)
C4—C5—C6—N1	−59.89 (15)	C22—C23—C24—C25	−0.9 (3)
C19—C5—C6—N1	172.82 (12)	C23—C24—C25—C26	−0.1 (3)
C4—C5—C6—C7	177.03 (11)	C22—C21—C26—C25	−2.1 (3)
C19—C5—C6—C7	49.75 (17)	O3—C21—C26—C25	−178.08 (19)
N1—C6—C7—C12	−58.45 (18)	C24—C25—C26—C21	1.6 (3)
C5—C6—C7—C12	63.83 (17)	C13—C2—N1—C27	59.84 (15)
N1—C6—C7—C8	124.20 (15)	C3—C2—N1—C27	−177.40 (12)
C5—C6—C7—C8	−113.52 (15)	C13—C2—N1—C6	−179.61 (12)
C12—C7—C8—C9	0.1 (2)	C3—C2—N1—C6	−56.85 (15)
C6—C7—C8—C9	177.50 (15)	C7—C6—N1—C27	−56.06 (16)
C7—C8—C9—C10	0.1 (3)	C5—C6—N1—C27	−178.67 (13)
C8—C9—C10—C11	−0.3 (3)	C7—C6—N1—C2	−176.10 (12)
C9—C10—C11—C12	0.3 (3)	C5—C6—N1—C2	61.28 (15)
C8—C7—C12—C11	0.0 (2)	C3—C4—N2—O1	3.7 (2)
C6—C7—C12—C11	−177.46 (14)	C5—C4—N2—O1	−178.11 (12)
C10—C11—C12—C7	−0.1 (3)	O2—C20—O1—N2	13.6 (3)
N1—C2—C13—C14	62.89 (18)	O3—C20—O1—N2	−169.00 (13)
C3—C2—C13—C14	−61.21 (19)	C4—N2—O1—C20	−174.36 (14)
N1—C2—C13—C18	−116.10 (16)	O2—C20—O3—C21	−1.9 (3)
C3—C2—C13—C18	119.80 (17)	O1—C20—O3—C21	−179.34 (15)
C18—C13—C14—C15	1.5 (3)	C22—C21—O3—C20	160.61 (16)
C2—C13—C14—C15	−177.54 (15)	C26—C21—O3—C20	−23.1 (3)
C13—C14—C15—C16	0.0 (3)		

### Hydrogen-bond geometry (Å, º)

**Table:**

*D*—H···*A*	*D*—H	H···*A*	*D*···*A*	*D*—H···*A*
C3—H3*A*···O1	0.97	2.27	2.6881 (18)	105
C26—H26···O2	0.93	2.30	2.823 (2)	115
